# The Effect of Protein–Starch Interaction on the Structure and Properties of Starch, and Its Application in Flour Products

**DOI:** 10.3390/foods14050778

**Published:** 2025-02-25

**Authors:** Jiayan Zhang, Yuhao Liu, Ping Wang, Yansheng Zhao, Ying Zhu, Xiang Xiao

**Affiliations:** School of Food and Biological Engineering, Jiangsu University, Zhenjiang 212013, China; jiayanzhang1988@163.com (J.Z.); 2212218020@stmail.ujs.edu.cn (Y.L.); l953420318@163.com (P.W.); zhaoys@ujs.edu.cn (Y.Z.); ying307@126.com (Y.Z.)

**Keywords:** starch, protein, starch–protein interaction force

## Abstract

Grains are an energy source for human beings, and the two main components—starch and protein—determine the application of grains in food. The structure and properties of starch play a decisive role in determining processing characteristics, nutritional properties, and application in grain-based foods. The interaction of proteins with starch greatly affects the structure, physicochemical, and digestive properties of the starch matrix. Scientists have tried to apply this effect to create foods tailored to specific needs. Therefore, studying the effect of protein on the structure and properties of starch in the starch–protein complexes will help in designing personalized and improved starch-based food. This paper reviews the latest research about the effects of endogenous and exogenous proteins on the structure and properties of starch, as well as factors influencing the interaction between protein and starch. This includes investigations of the chain and aggregation structure of proteins with starch, as well as assessments of impacts on thermal properties, rheology, gel texture properties, hydration properties, aging, and digestion. In addition, particular examples illustrating the effects of protein–starch interaction on starch properties in various foods are discussed, providing a reference for designing starch–protein foods that are rich in terms of nutrition and easier to process.

## 1. Introduction

Cereals and grains are key dietary components, and they dominate most food-based dietary recommendations to achieve intake goals in terms of carbohydrates, protein, and dietary fiber [1,2,3]. Starch is the main component of grains, and starch’s structure and properties are of great significance to the processing and nutritional properties of grain-based foods [4,5]. Researchers have carried out Pearson correlation analysis on various parameters of grain starch structures (including starch chain structure, aggregated structure) and starch properties (gelatinization, gel texture properties, enzyme hydrolysis, etc.) before. They have found that starch’s composition and internal structure were significantly correlated with various starch physicochemical properties (*p* < 0.05). For example, the relative crystallinity, amylopectin content, and amylose content of starch were strongly correlated with its gelatinization properties, including swelling potential, solubility, gelatinization temperature, and viscosity [6,7,8,9]. The chain length distribution of amylopectin is closely related to the gel texture properties of starch. Furthermore, it had been found that the short-range molecular order, relative crystallinity, and ratio of amylopectin and amylose of starch were closely related to the in vitro digestibility of starch [9].

At the same time, the effects of the non-starch components of cereals on the structure and physicochemical and digestive properties of starch have also been extensively studied. The results of some of the studies show that the non-starch components of cereals (polyphenols, polysaccharides, lipids, and proteins) have a significant impact on the nutritional properties of cereal products [10,11,12,13]. The control of the microstructure in food mixing systems is critical for determining product characteristics, including organoleptic properties, nutritional properties, and processing requirements [14,15,16]. Of all non-starch components, protein removal has been found to have had the greatest impact on starch’s structure, physicochemical properties, and digestive properties [17,18]. The removal of proteins significantly impacts starch by altering its structure, increasing swelling capacity and water absorption, modifying gelatinization and pasting properties, and enhancing digestibility due to reduced protein–starch interactions. Thus, protein, the second largest biopolymer component of cereals crops, not only provides nutrients and energy needed by the human body, but also has an important impact on the structure [19,20], physicochemical properties [21], and digestive properties of starch [22,23]. More and more researchers have begun to explore protein-related mechanisms in food mixing systems that affect the food matrix. Through this avenue, the beneficial effects of starch–protein interactions can be exploited to find improvements that add to the benefits of specific starchy foods.

In general, proteins directly or indirectly affect the structure and properties of starch through their interactions with starch, thereby affecting the quality and nutritional properties of starch-based products to varying degrees. Starch–protein interactions are affected by starch-based food processing conditions (ionic strength, pH), sample pretreatment (fermentation, extrusion), and other additives (chitosan and citric acid). For example, the relative crystallinity of protein–starch mixtures can be improved by exogenously adding specific types of proteins [24]. This effect may be used to prepare different foods with specific traits and nutritional properties.

This review aims to highlight various types of interactions between endogenous and exogenous proteins and starch, and the factors that influence such interactions. The effects of starch–protein interactions on the structure, physicochemical properties (gelatinization, rheological properties, gel texture, aging recovery, hydration, etc.), and digestive properties of starch are reviewed. In addition, the effects of adding specific proteins to starch-based foods (such as dough, bread, steamed bread, noodles, and rice flour) are covered. Thus, it serves as a reference for developing cereal-based foods with improved starch substrates, better properties, and enhanced nutritional value.

## 2. Starch–Protein Interaction Force and Factors Affecting Starch–Protein Interactions

### 2.1. Starch–Protein Interaction Force

Protein is the second largest macronutrient in grains after starch, and its content ranges from 8 to 40%, depending on the plant species and growing environment. Together with starch, it is encapsulated by the endosperm cell wall [14,25]. Grain endogenous proteins are mainly divided into two types: storage proteins and starch granule-associated proteins, as well as granule-associated proteins (SGAPs) [26,27]. In cereals, the main proteins are storage proteins, which provide nutrition for embryo growth and maturation. During the isolation of starch from cereals, starch still contains 0.1–0.8% residual proteins that are difficult to remove, known as starch granule-associated proteins (SGAPs) [14]. Unlike storage proteins in cereals, starch granule-associated proteins tightly bind to the surface (starch granule surface proteins, SGSPs) or internal channels (starch granule channel proteins, SGCPs) of starch granules [28,29]. This close physical association of proteins with starch produces an interaction that has a major impact on the properties of the starch [30]. Consequently, elucidating these interaction mechanisms provides critical insights for rationally designing starch-based food systems

**Figure 1:**
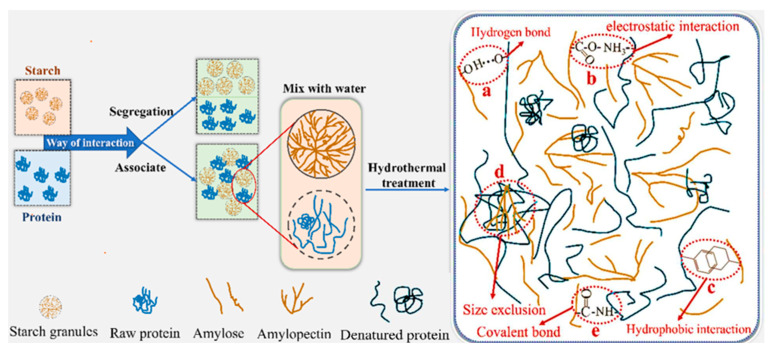
Starch–protein interactions.

The interactions between protein and starch can result in two distinct states: segregation and association. Segregation refers to the phase separation between protein and starch, where the two components exist as distinct phases. In contrast, association describes a state of relatively uniform mixing, where protein and starch are more homogeneously distributed. These states significantly influence the structural and functional properties of the system [31]. The main interaction forces are as follows: covalent bonds, electrostatic interactions, hydrogen bonds, ionic bonds, hydrophobic interactions, and size exclusion (as shown in Figure 1).

Regarding the non-covalent interaction between protein and starch, Zhang et al. [21] studied the interaction of endogenous maize proteins (i.e., starch granule-associated proteins and storage proteins) with starch binding, and showed that starch granule-associated proteins (SGAPs) interact with starch chains through hydrogen bonding, hydrophobic interactions, and electrostatic interactions. SGAPs have a strong binding effect on starch, while storage proteins only interact through hydrogen bonds. Huang and Lu et al. [32,33] showed by Fourier transform infrared spectroscopy (FT-IR) analysis that the intermolecular hydrogen bonds of Inca peanut seed albumin, corn starch, rice protein, and rice starch are mainly interaction forces. The interaction between protein and starch may also be an electrostatic interaction, since starch has negatively charged phosphate groups, whereas proteins may be positively charged [34,35]. Wang et al. [24] observed the rheological properties of indica rice starch-mixed systems with different protein types. They found that the interaction between indica rice starch and whey protein and casein is a hydrophobic molecular interaction, and the interaction with soybean protein isolate involves hydrophobic, hydrogen bonding, and electrostatic interactions. Wu et al. [36] compounded starch modified with whey protein and octenyl succinic anhydride and found that, when the compound’s pH was 4.5, the modified starch of octenyl succinic anhydride and whey protein complexed to form a smaller-particle-size soluble complex. They proposed that the main interaction force of this complex is a hydrophobic interaction. Enzyme-treated potato protein and potato starch-mixed gel has strong hydrophobicity, hydrogen bonding, and electrostatic forces, which can form a strong co-gel network structure [37]. Under static attraction and electrostatic rejection, an ion bond is formed when the two are balanced, but there are very few reports about starch–protein direct ion bonds.

In addition, regarding the covalent interaction between protein and starch, since starch molecules contain terminal reducing groups, the Maillard reaction is considered to be the most common covalent binding reaction between starch and protein in starch-processed food systems, often producing attractive aromas, colors, and flavors in baked products [38,39]. Covalent and hydrogen bonding are the main interactions for adding high-gliadin/glutenin starch dough [40]. Li et al. [41] studied the interaction of A/B-type starch with gluten. The results showed that the main force of the starch–gluten interaction was a covalent bond (Sulfhydryl), that the main force of the starch–gluten mixing stage was hydrophobic interaction, and that hydrogen bonds and covalent bonds were involved in the interaction at the starch–gluten mixing stage.

### 2.2. Factors Affecting Starch–Protein Interactions

**Figure 2:**
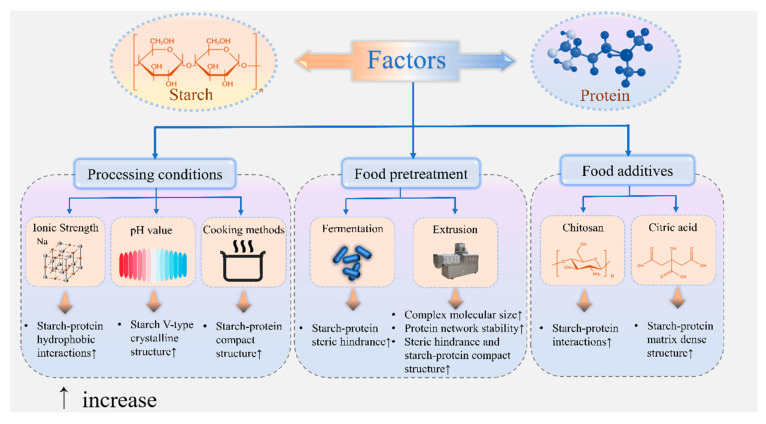
Factors affecting starch–protein interactions.

In addition to changes caused by interactions between food components themselves, food properties are also affected by food processing methods (e.g., extrusion, milling, and drying), cooking techniques (e.g., boiling, steaming, and baking), and pretreatment steps (e.g., soaking, fermentation, and enzymatic treatment) [42,43,44,45,46]. These processes influence the interactions between starch and protein in food matrices, and studying their effects can help to guide the formulation and processing conditions for creating starch–protein-mixed foods with specific properties (as shown in Figure 2).

#### 2.2.1. Processing Conditions

Studies have shown that starch–gluten dough gelatinization is inhibited in the presence of NaCl. This is possibly because, in the presence of NaCl, the binding of salt ions (Na^+^ and Cl^−^) to the hydrophilic groups of proteins in the mixed system promotes the binding of protein–protein or protein–starch molecules by hydrophobic interactions. While promoting the formation of a gluten protein network structure, it also enhances the hydrophobic interaction of starch and protein, thereby inhibiting starch gelatinization [47,48].

Since starch-based foods contain a wide range of pH values, studying changes in starch–protein interactions at different pH values during starch food processing can help to guide the design of different types of foods that meet target requirements. Bravo et al. [49] studied changes in the physicochemical properties of corn starch mixed with vegetable proteins (pea and rice) and animal proteins (albumin and whey) in equal proportions at different pH values (4.5, 6.0 and 7.5). They observed increased gelatinization temperatures under acidic pH (4.5) values. This was likely due to the hydrolysis of amorphous starch regions generating low-molecular-weight dextrins, which reduced the destabilization of crystallites and delayed gelatinization. The starch–whey mixture exhibited maximum hardness at a pH of 7.5, which was attributed to pH-dependent protein aggregation near the isoelectric point (pI), where reduced electrostatic repulsion enhances hydrophobic interactions and particulate gel formation. From this, it can be inferred that pH may have an effect on starch–protein interactions, leading to changes in the physicochemical properties of the mixtures. Zhen et al. [50] studied changes in the structure and digestive properties of whey protein isolate–indica rice starch–stearic acid at different pH levels. They attributed the pH-dependent structural changes to the weakening of the network structure and increased nonperiodic domains to higher pH levels. These alterations hindered enzyme diffusion and access to starch chains. Additionally, whey protein acted as a physical barrier, blocking amylase binding sites and inhibiting enzymatic activity, thereby enhancing starch resistance to digestion. Xuemin et al. [51] investigated the effects of different cooking methods (steaming, boiling, and baking) on the structure and digestive properties of the gluten–wheat starch–lauric acid complex. The results show that the microstructure of the cooked and baked samples is denser, accompanied by an increase in hydrolysis resistance. Therefore, different cooking methods showed a significant effect on the compact structure of starch-based foods composed of complex ingredients.

#### 2.2.2. Food Pretreatment

Villanueva et al. [52] conducted a study to evaluate the impact of microwave treatment (MWT) on products formulated with potato and rice starch supplemented with 5% calcium caseinate (CaCAS) or soy protein isolate (SPI). The findings demonstrated that applying MWT to protein–potato blends improved gel stability, reduced their pasting profiles, and led to increased viscoelastic moduli. Navneet et al. [53] discovered that bean flour underwent distinct temperature treatments—dry heat (DH) and extrusion—which significantly altered the microstructure and molecular architecture of its starch and proteins. DH processing primarily affects protein conformation while minimally impacting starch structure. In contrast, extrusion leads to extensive structural modifications in both starch and protein. Both DH and extrusion treatments reduced the intramolecular β-sheet secondary structure of bean protein. These heat treatments not only caused color variations but also influenced particle size distribution, pasting profile, rheological properties, and water absorption and solubility indices of the bean flour. Buksa et al. [54] studied the interaction of modified arabinoxylan–starch–protein in rye dough during fermentation. The results of HPSEC showed that the complex composed of starch–cross-linked arabinoxylan–rye protein had the highest molecular size. A decrease in the HPSEC signal after fermentation represents the formation of complex precipitates. Thus, fermentation leads to a more compact formation of starch–polysaccharide–protein ternary complexes. Téllez-Morales et al. [55] investigated the structural changes of cornstarch and whey protein isolate blends during extrusion. It was found that there was a significant corresponding change in the blend structure relative to the mathematical model of extrusion (*p* < 0.05). This model is therefore useful for predicting changes in cornstarch–whey protein isolate interactions.

#### 2.2.3. Food Additives

Yang et al. [56] investigated the effect of weight chitosan (CHT) on the interaction of whey protein isolate (WPI)–native wheat starch (WS). Since the CHT side chain contains hydroxyl and amino groups, the addition of CHT can promote intermolecular interactions in the ternary system, thereby increasing the storage modulus (G′) of the mixture. Compared with the starch–protein mixture without pectin addition, the viscosity of the system increased with the addition of pectin, indicating that there was a strong interaction between the starch–protein network and pectin. In addition, it was found that pectins with higher side-chain lengths and higher molecular weights interact more strongly with starch–protein matrices, which helps to improve the rheological properties of starch–protein doughs [57]. Zhu et al. [58] observed that Arabian xylan–starch–gluten protein interactions occurred within the gluten network, facilitating enhanced contact and connectivity between starch and gluten protein. This interaction contributed to improved dough extensibility and deformability. Zeineb et al. [59] conducted a study comparing the impact of rapeseed oil and palm oil on cakes during storage. They discovered that starch retrogradation increased the tryptophan fluorescence intensity of gluten protein over time. This effect was attributed to the quenching of hydrogen bonds between amylose and gluten protein, caused by the addition of oil. Notably, cakes containing palm oil exhibited higher fluorescence intensity compared to those with rapeseed oil.

Although there have been relatively few studies on the factors affecting starch–protein interactions, consideration should be given to processing conditions, sample pretreatment, and the effects of food additives when studying complex food interactions. The complexity of the food system and different processing conditions will affect the properties of protein-containing starch-based foods [48,60,61,62].

## 3. Effects of Starch–Protein Interactions on Starch Structure, Physicochemical and Digestive Properties

### 3.1. Effects on Starch Structure

The influence of protein on grain starch can be described by considering several factors. This includes the starch chain structure, the content and proportion of amylose and amylopectin, the amylopectin chain length distribution, the aggregation state, and the starch molecular weight size and distribution. Structure refers to the short-range molecular order, the helical structure, the crystal structure, the lamellar structure, and the starch granule structure [63]. At present, the research on the influence of protein on starch structure mainly focuses on the aggregated structure of starch, such as the long-range ordered structure of starch, as characterized by X-ray diffraction (XRD), and the short-range molecular ordered structure of starch, as characterized by Fourier transform infrared spectroscopy (FTIR) [24]. The research on the influence of starch properties includes thermal/pasting properties, rheological properties, gel texture properties, hydration properties, and aging properties [64].

#### 3.1.1. Effects on Starch Chain Structure

The number of short and long chains of starch was reduced, and larger colloidal clusters were produced when Wang et al. [24] added protein to starch while cooling after thermal processing. Some of the proteins also formed a more continuous gel network structure with starch after gelatinization. Lu et al. [64] measured the short-range molecular order and starch recrystallization of potato starch with added potato protein, and found that the potato protein helped to inhibit the expansion of starch granules during the heating and gelatinization of starch, and the hydrogen bond between protein molecules and starch molecules promotes the rearrangement and crystallization of starch molecules, thus promoting the recrystallization of amylopectin during cold storage. Recrystallization occurred during the process to inhibit the digestion of starch. It is possible that the protein promotes the relative crystallinity of starch by increasing the interaction between amylopectin chains and further inhibiting the recrystallization of starch molecules [22].

#### 3.1.2. Influence on the Structure of Starch Aggregates

The aggregated structure of starch is influenced by the degree of its chain structure, which is why research on the impact of protein on starch has primarily focused on the aggregated structure. Li et al. [19] prepared mixtures of potato protein modified with laccase or tyrosinase and potato starch in different proportions, and the relative crystallization rate of the mixtures obtained increased. Similar findings were also found between whey protein and oat starch. XRD and FTIR studies showed that the addition of whey protein would improve the ordered structure of oat starch granules and significantly increase its relative crystallinity [65]. When Qian et al. [66] selectively removed starch SGCPs and SGAPs, the removal of both reduced the apparent crystallinity of starch, and the former had a significant effect on the structural properties of starch. The impact of GCP removal was more significant than the removal of SGAPs. Endogenous protein can increase the relative crystallinity of adlay seed starch, which indicates that the endogenous protein in adlay seeds promotes the tight binding of the aggregated structure of the starch structure [67]. Similarly, the addition of exogenous protein increases the starch crystallinity of recombinant rice [68].

Many scientists have found that the addition of amino acids will also have beneficial effects on the structure, gelatinization, and digestion of starch [69]. The addition of cysteine increases the relative crystallinity of glutinous rice starch. The effect of adding corn starch to amino acids was observed by Ji et al. [70] through a polarizing cross microscope, and they found that the birefringence brightness of corn starch granules became lower after treatment, which also confirmed that the relative crystallinity of corn starch after treatment was reduced.

### 3.2. Influence on Physicochemical Properties and Possible Reasons

Endogenous and exogenous proteins can limit the hydration, gelatinization, and viscoelasticity of millet starch granules by interacting with starch. This can further promote the retrogradation of starch molecules in grains and reduce the digestive properties of starch [27,71,72].

#### 3.2.1. Thermal Characteristics and Pasting Properties

Differential scanning calorimetry (DSC) and rapid viscosity analysis (RVA) are available to assess the thermal characteristics and pasting properties of substances, respectively. Because starch is a macromolecular polymer polymerized from glucose by glycosidase, DSC can reflect its thermal characteristics, including initial gelatinization temperature (To), gelatinization peak temperature (Tp), gelatinization end temperature (Tc), and gelatinization enthalpy (ΔH). An RVA (Rapid Visco Analyzer) was used to determine viscosity profiles of starch, including pasting properties like peak viscosity (PV), trough viscosity (TV), final viscosity (FV), breakdown (BD), and setback (SB) values).

There are two research perspectives on the influence of protein on the thermal characteristics and pasting properties of starch: the exogenous addition of protein (or peptide and amino acids) and the removal of endogenous protein from grains.

##### Direct Exogenous Addition of Protein

As a high-quality biological protein, whey protein isolate (WPI) has the characteristics of enhancing water-binding capacity and easily forming a three-dimensional network structure when the protein chain unfolds during denaturation [73]. Researchers often exogenously add WPI to grain starch to try to improve the thermal properties of starch. Studies have shown that the exogenous addition of WPI leads to an increase in the viscosity of hot oat starch paste, and a decrease in relative breakdown. Whey protein has a significant effect on oat starch by increasing the peak temperature of DSC [65]. The results of RVA showed that the interaction between whey protein and starch accelerated the swelling of starch granules and inhibited the swelling and gelatinization of starch, thereby helping to reduce the thermal stability of a starch gelatinized body, casein. In contrast, the interaction between casein and starch exhibited the opposite effect, enhancing thermal stability [24,74]. The addition of albumin fraction (IPA) resulted in a significant increase in the thermal properties (To, Tp and Tc) of corn starch, but there was no significant change in ΔH. Here, the interaction between the IPA molecules and the corn starch molecules possibly hindered the water-binding properties of the starch molecules, resulting in a higher temperature requirement for starch molecules to gelatinize [32]. Wang et al. [68,75] studied the effect of the addition of different amounts of rice protein on the thermal characteristics and pasting properties of recombinant rice flour, and found that different protein additions (0–8%) would reduce the gelatinization peak viscosity of recombinant rice flour by 444 centipoise (cp) and increase the gelatinization temperature by 3.2%. Similarly, the exogenous addition of glutathione and glutenin inhibited the swelling and gelatinization of wheat starch by interacting with wheat starch [76].

##### Exogenous Addition of Enzyme-Modified Proteins or Amino Acids

Many studies have shown that the hydrolyzed protein has a greater impact on the properties of starch than the original protein [77,78]. The possible reason for this is that the protein chain expands after hydrolysis, resulting in the leakage of the hydrophilic group, which competes for the moisture necessary for starch gelatinization. Amino acids and peptides, as by-products of grain processing, were also added to starch to study their effect on starch properties. Lin et al. [22] found that rice protein hydrolyzate limited the hydration swelling of starch and decreased the ΔH, while increasing the gelatinization temperature. Likewise, the PV, TV, and FV of the enzymatically treated blends of potato protein and potato starch were reduced by 12.7–41.1% [19]. Li et al. [79] added glutamic acid to starch to simulate the effect of starch–protein interactions on the gelatinization properties of japonica rice starch. They found that the starch gelatinization property index was significantly correlated with protein structure (carbonyl content, disulfide bond content) (*p* < 0.05), which indicated that glutamic acid could affect starch gelatinization properties by interacting with starch. The glutelin between starch granules could form a network structure by hydrogen bonding with starch to inhibit gelatinization. Li et al. [69] measured the gelatinization characteristics of corn starch with amino acids added, and found that the gelatinization temperature of corn starch increased and the ΔH decreased after amino acids were added. The gelatinization viscosity of potato starch decreased after adding enzyme-modified protein, but To, Tp, Tc, and ΔH were not significantly different compared with pure potato starch (*p* > 0.05) [80].

Kong et al. [81] investigated the effect of porcine plasma protein hydrolyzate on the gelatinization properties of cornstarch. They found that the gelatinization temperature of cornstarch increased significantly (*p* < 0.05) with the addition of protein, and the PV, FV, and SB values were significantly reduced compared with examples with no protein component. According to a microstructural analysis of starch using a confocal laser scanning microscope (CLSM) and an atomic force microscope (AFM), it was shown that the porcine plasma protein hydrolyzate can physically encapsulate corn starch, and so the protein has an inhibitory effect on the gelatinization of corn starch. The addition of different amino acids, especially charged amino acids such as Glu and Lys, can inhibit the swelling and gelatinization of starch granules during heating, thereby delaying the entry of water into the interior of starch molecules, further reducing the PV, TV, FV, and SB values of starch [82].

##### Endogenous Protein Removal to Verify the Effect of Protein on Starch Thermal Properties

Compared with the method of adding exogenous protein to verify the influence of protein on starch properties, it has been shown that the endogenous protein of many grains itself has a protective effect on the properties of grain starch [17,66,83]. The research is shown in Table 1. According to these studies, the effects on starch properties of different types of endogenous grain proteins varies, but in general, endogenous grain proteins have a protective effect on starch granules. As Baldwin et al. [84] found that SGAPs may act as a kind of boundary membrane, and if this boundary membrane is removed, the starch granules are more likely to absorb water and facilitate the gelatinization of starch granules under conditions where the energy input exceeds the gelatinization temperature. In addition to this, they found that, although potatoes contained very little endogenous protein, removing potato protein had a large effect on the properties of potato starch. Thus, grain protein is one of the factors affecting the properties of starch, and its potential dose–effect relationship merits further exploration.

#### 3.2.2. Rheological Properties of Starch–Protein Systems

The instrumental measurement of rheological properties plays an important role in product quality prediction. Knowing the correlation between the rheological properties of a product and its approximate chemical composition and sensory properties helps to evaluate its technical and edible quality [88,89,90,91,92]. For example, the rheological properties of starch not only predict changes in starch-based food due to food processing, quality control, and storage stability, but also provide insights into starch gelatinization, retrogradation, and interactions with other food components [93,94]. The rheological properties of starch are not only affected by the structure of starch itself, but also by non-starch substances, such as proteins in starch-based foods, and so it is necessary to study the effects of proteins on the rheological properties of starch. The commonly measured rheological properties of starch are measured by examining its steady-state rheology and dynamic rheology, or more specifically, oscillatory frequency and steady shear flow, respectively [93]. Through an oscillation frequency test in the range of −10Hz, the storage modulus (G′) and loss modulus (G″) of starch paste can be obtained. The steady shear flow is the curve of the shear stress of the sample versus the shear rate, at about 0.01–300s^−1^, from which the hydrodynamic type of the starch sample can be obtained (solid, Newtonian, or non-Newtonian fluid).

Regarding the exogenous addition of protein, studies have shown that the storage modulus values of enzyme-treated protein and potato starch blends are lower than those of natural blends [19]. Steady-state shear rheological tests showed that, when the concentration of peanut seed albumin fraction was 5%, the consistency coefficient (K) value of the protein starch system was the largest. And the storage modulus (G′) and loss modulus (G″) of corn starch after protein addition were higher than in cases without protein addition [32]. Li et al. [40] investigated the rheological effect of the interaction between gliadin and glutenin and starch on the mixed system. They found that the effect of the gliadin/gluten ratio on dough elasticity and strength was dependent on the mixing stage of the dough: increasing the ratio in the optimal mixing stage reduced the stability of the dough pattern, while other stages promoted the network structure of the mixture. The G′ and G″ values of the protein starch mixture were the smallest at 9% gluten concentration [95]. Bravo et al. [96] studied the effect of the addition of different proteins (potato, rice, pea, and hydrolyzed gluten) on the rheological properties of protein–cornstarch mixtures. They found a decrease in G′ and an increase in G″ for all protein and flour mixtures, indicating that the addition of protein shifts the cornstarch network from displaying elastic to viscous properties. After adding the enzyme-modified protein to potato protein, the apparent viscosity of starch decreased with an increase in shear rate, that is, the phenomenon of shear thinning occurred. At the same time, the G′ and G″ of starch increased [80].

Regarding the effect of grain endogenous protein on the rheological properties of grain starch, the study also showed that grain endogenous protein had a significant effect. The removal of SGAPs for rice and small-granule starch weakened pseudoplasticity and shear-thinning behavior [83,97]. The removal of SGAPs exerts divergent effects on the rheological properties of various starch types. Specifically, in the case of waxy starch, the elimination of SGAPs leads to a reduction in the elasticity of the three-dimensional network structure within the gel, thereby weakening the overall gel matrix. Conversely, for non-waxy starch, the absence of SGAPs results in an increase in gel structure elasticity and an enhancement of the gel network’s strength [97].

#### 3.2.3. Gel Texture

Starch–protein interactions alter the multiscale chain reorganization of gelatinized starch into different structures during cooling. For example, Wang et al. [24] found that indica rice starch, whey protein, and soy protein isolate can form a larger gel network, while indica rice starch with casein forms a more continuous gel network. After gluten was added to starch, scanning electron microscopy (SEM) and texture analysis found that the addition of protein could reduce the hardness, chewiness, and gummy viscosity of bread by inhibiting the leaching of starch, improving the product quality of gluten-free bread [95]. The addition of whey protein concentrate reduces the hardness, cohesiveness, adhesive force, and adhesiveness of wheat starch gel. It weakens the network structure of wheat gel, while it also inhibits the retrogradation of wheat starch. Therefore, it can actively improve the storage characteristics of wheat starch [98]. The addition of protein inhibits the retrogradation and water migration of rice flour gel by binding water more strongly than starch molecules, thus maintaining a uniform water distribution and a moderate gel shape. This is attributed to the protein’s ability to reduce the collision probability between gelatinized starch molecules, thereby suppressing the formation of ordered structures during retrogradation [68]. After adding soy protein isolate, CLSM and texture analysis showed that the hardness, chewiness and gumminess of gluten-free bread decreased, thus improving the quality of gluten-free bread [95].

#### 3.2.4. Hydration Capacity—Solubility and Swelling Power

Starch granules are composed of amylose and amylopectin formed by the polymerization of glucose through glycosidic bonds [99]. Most of the amylopectin chains are intertwined with right-hand double helixes to form a dense crystalline structure, but most of the amylose regions can only form a starch amorphous region [99]. The essence of starch gelatinization is that, when the temperature is higher than the gelatinization temperature, the starch granules first absorb water and expand from the amorphous region. The crystallization region then collapses, and the whole starch forms a uniform paste-like system [100,101]. During gelatinization, non-starch components often protect the granules, resulting in resistance to water absorption and swelling. As protein is the second largest nutrient component of starch-based foods, its impact on hydration—measured via the water solubility index (WSI) and swelling power (SP)—should not be underestimated. Relevant studies have shown that the removal of rice granule-related proteins reduces the thermal stability of rice starch, thereby increasing the leaching, swelling power, and solubility of rice amylose [83]. Whey protein concentrate supplementation reduces the swelling power of wheat starch [98], and whey protein concentrate reduces the swelling and viscosity of native wheat starch gels [98]. Bravo investigated the effect of the addition of different proteins (potato, rice, pea and hydrolyzed gluten) on the hydration capacity of flour. They found that the addition of rice and pea protein significantly enhanced the hydration capacity of flour, and the increased strength was positively correlated with protein content; among all proteins, pea protein had the strongest enhancement effect on flour hydration, while potato protein had the weakest effect [96].

#### 3.2.5. Aging Properties

The aging of gelatinized starch is the main reason for the decline in the quality and shelf life of starch in food [102]. The whey protein concentrate has a significant delaying effect on the retrogradation of native wheat starch, that is, it can improve the functional properties of starch gel [98]. At a certain addition ratio (10%), whey protein concentrate can significantly reduce the recovery rate of natural wheat protein, indicating that adding an appropriate amount of protein can effectively improve the functional properties of the gel [98]. Hu et al. found that whey protein hydrolyzate can be used as an additive to inhibit the aging of rice products and can also enrich the nutritional function of the product [103]. The exogenous addition of glutathione and gluten can delay the aging of wheat flour dispersions. The main reason is that gluten and glutathione are embedded in the wheat starch chain, which can inhibit the aging of wheat starch by inhibiting the rearrangement of amylose [76]. Niu et al. [104] investigated the effect of porcine plasma protein hydrolyzate on the aging properties of cornstarch. They found that the addition of porcine plasma protein hydrolyzate reduced the setback viscosity of cornstarch, which helped to inhibit cornstarch retrogradation. Combined with the XRD pattern, the addition of porcine plasma protein hydrolyzate can interact with corn amylose to change the structure of amylose in a way that impacts aging, thereby inhibiting corn starch aging. Some studies conclude the opposite, that the addition of soy protein accelerates the aging of corn starch [105]. The addition of whey protein concentrate weakens the network structure of wheat starch gel by reducing the hardness, cohesiveness, adhesive force, and adhesiveness of the wheat starch gel. However, it has an inhibitory effect on the retrogradation of wheat starch and can actively improve wheat starch [98].

#### 3.2.6. Effects on Digestion (Hydrolysis) Properties

Starch is the most important macromolecular polymer for human energy acquisition [106]. Its digestive properties in the human body have garnered interest, especially in recent years, due to the increasing prevalence of chronic diseases caused by obesity, blood sugar, and lipid metabolism syndrome [107,108]. Since starchy foods are human staple foods, the digestibility of starch is a concern [109,110,111]. In order to easily identify the digestive properties of starch, researchers have categorized starch as rapidly digestible starch (RDS), slowly digestible starch (SDS), and resistant starch (RS) according to the starch digestion time [99,112,113]. The content of RDS is proportionally related to the glycemic index (GI). A higher content of starch will be more conducive to the production of low-GI food. However, both endogenous and exogenously added grain proteins can interact with starch granules in various intermolecular ways to prevent them from being invaded by α-amylase. Thus, the type and content of non-starch components in the food matrix, such as proteins, can be used to control starch’s digestibility appropriately.

Research related to the influence of endogenous protein on the digestion characteristics of grain starch is shown in Table 2. Table 2 shows that current research relies mostly on methods that remove endogenous protein directly using protease removal. Some studies have further refined the types of proteins, for example, focusing on the removal of SGAPs and storage proteins. The research on the influence of exogenous added protein on starch digestion characteristics is shown in Table 3. Most of the researchers directly added high-quality vegetable protein [22,114,115,116]. However, since it was discovered that the mechanism of protein inhibition of starch digestion stems from the improvement of hydrogen bonds between protein and starch, researchers have gradually added the hydrolyzate hydrolyzed by protease to starch. Compared with the direct addition of the original protein, this hydrolyzate has a more obvious inhibitory effect on starch digestion [22,117]. For example, the exogenous addition of protein confirmed that the influence of protein on starch digestion characteristics is positive. This suggests that protein–starch interactions are not limited to the use of natural proteins.

Thus, a variety of research methods have shown that the endogenous protein of the grain exerts differing degrees of inhibition on starch digestion characteristics. The mechanisms can be categorized as follows: (a) grain proteins form a physical barrier between starch and α-amylase; (b) the interaction of proteins with starch promotes the formation of starch-resistant compact structures; (c) the interaction between proteins and α-amylase limits the exposure of starch to amylase. These results indicate that both endogenous and exogenously added proteins interact with starch and they play an important role in reducing starch digestibility. These findings contribute to the development of low-glycemic-index starch foods through the compounding of plant proteins.

## 4. Applications of Starch–Protein Mixture in Starch Food

**Figure 3:**
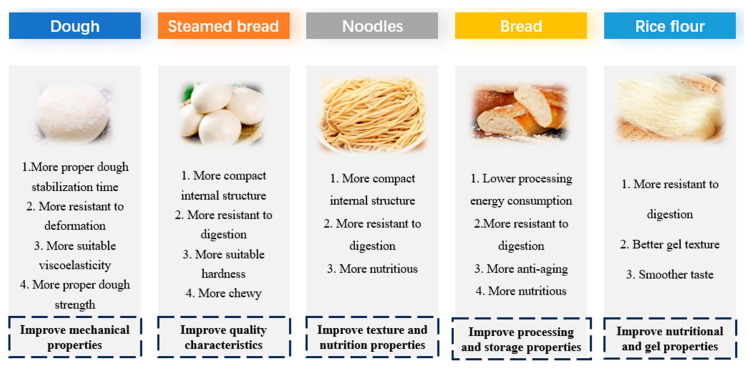
Application of protein–starch mixtures in starchy food.

Previous studies have shown that the pasting and thermal properties of starch affect the cooking quality of grains [124,125,126]. The aging properties of starch affect the texture of cooked grains during storage [127]. The gel texture of starch determines the finished texture and organoleptic properties of starch-based foods [128]. Protein helps inhibit the alpha-amylase digestion of starch in noodles [23,117,129]. Therefore, the addition of specific types and contents of protein can reduce the digestion of starch, thereby contributing to the development of starchy foods with tailored functional properties. Overall, the physicochemical and digestive properties of starch are critical to its ultimate utilization. In particular, they have specific effects on starch food substrates, and have guiding significance for the cultivation of specific varieties (grains with low starch digestibility). Some studies have now exploited the beneficial effects of protein on starch, as applied to foods such as dough, steamed bread, bread, noodles, and rice flour (as shown in Figure 3).

### 4.1. Dough and Steamed Bread

In order to optimize the mechanical properties (deformation resistance, dough strength) of protein-added dough and the quality of steamed bread (affordability, firmness), researchers have tried different protein types and addition ratios, with good results [130,131,132].

Li et al. [133] compared the thermal properties, rheological properties, and quality of steamed bread with different varieties of wheat flour and gluten–starch dough. The results showed that the stability time and gelatinization viscosity of gluten–starch dough were higher and more stable than those of wheat dough. As a result, steamed bread made with gluten–starch dough is more chewy.

In order to obtain gluten-free dough with better viscoelasticity, Tandazo et al. [134] systematically studied the rheological behavior of different types of proteins and different types of starch systems. It was finally found that rice starch could improve the deformation resistance and dough strength of corn gluten dough. This study found the interaction of specific proteins with specific starches helps to develop gluten-free doughs with suitable viscoelastic properties.

Li et al. [133] investigated the mechanical properties of steamed wheat bread dough and the quality of steamed bread under two protein addition modes, i.e., when the starch/gluten ratio was consistent with the original flour, and when the protein content was consistent with the original flour. The results showed that the model dough with the same starch/gluten ratio as the original flour had a better dough elongation performance, mechanical strength, and dough stability time, and the internal structure of the steamed bread was more compact. This indicates that a certain starch/gluten ratio improved the firmness and chewiness of the steamed buns.

### 4.2. Bread and Noodles

Studies have found that the addition of protein can optimize the nutritional, processing, and storage characteristics of starch-based products, including reducing starch digestibility, improving energy consumption, and improving the anti-aging properties of bread and noodles [35,135]. For example, adding laccase significantly (*p* < 0.05) decreased the starch in vitro digestibility and maximum hydrolysis extent of black barley noodles [114]. Hu et al. [95] inhibited the leaching of starch by adding gluten, reduced the hardness, chewiness and gumminess of the system, and improved the product quality of Gluten-free bread. The results showed that the addition of 12% soybean protein isolate could significantly (*p* < 0.05) reduce the ΔH and setback (SB) values of starch. The addition of protein helps to reduce the ability of the bread to heat up and makes it more resistant to aging. The starch digestion of Italian noodles, with broad bean protein added, was significantly (*p* < 0.05) reduced, and so broad bean protein could reduce the production of blood sugar while strengthening the nutritional function of the pasta [136]. Zou et al. [137] have studied the effects on starch digestion of removing protein from Italian pasta. Based on CLSM, it has been shown that complete pasta has lower starch digestion due to the wrapping of gluten protein. This is due the inhibition of pasting in the internal structure of the Italian pasta surface in the starch cooking process, making the internal structure of the Italian noodles more compact, which can inhibit the enzymatic effects of starch hydrolytic enzymes. Sissons et al. [115] have also studied the digestive impact of adding grain protein to pasta. They have found that the in vitro starch digestion of Italian noodles decreases with an increase in protein. Based on a micro-structure analysis, the addition of protein can make cooked pasta form a denser gel mesh structure, which helps restrict the invasion of amylase grain particles and reduce starch digestion.

Li et al. [23] have removed protein from high-amylose wheat powder, and have shown that the protein in high-amylose, high-straight-chain wheat powder has a suppressing effect on the digestion characteristics of wheat starch. This discovery can contribute to further strengthening the nutritional value of wheat as a staple product. Moreover, the addition of broad bean flour, concentrated protein, and separation protein helps to increase the slow digestive starch content of wheat starch in the pasta. The addition of protein can also improve the nutritional quality and physiological function of wheat noodle products [136]. Some studied the addition of fish protein to pasta and showed that the protein digestion of Italian noodles increased significantly after addition. At the same time, it also helped to enhance the toughness of the Italian pasta surface and reduce the paste of starch. Adding an appropriate amount of fish protein can ensure that Italian noodles do not produce an unacceptably fishy smell, while also increasing the nutritional function of Italian noodles and the energy consumption required for cooking [138]. Graca et al. [139] studied the impact on starch digestion of adding yogurt and cream cheese to small commercial bread. It was found that the RDS content was significantly (*p* < 0.05) reduced after the addition. The RS content also increased, which improved the low blood glucose index score of the bread.

### 4.3. Rice Flour

Studies have found that the addition of protein can help rice noodles maintain better gel texture properties by controlling the moisture migration of rice noodle gel [140]. At the same time, it can also have a certain inhibitory effect on the digestive characteristics of rice noodles. Khatun et al. [119] found that the addition of certain monomer rice proteins can help to reduce the starch digestion of rice noodles. This discovery helps in reducing the digestion of rice starch by cultivating rice with a specific protein content. In addition, the digestion of rice starch can be controlled quantitatively by adding specific proteins. The formation of the protein–starch matrix contributes to the formation of a rice flour paste with specific gelatinization properties. The starch–protein network structure can increase the viscosity of rice flour paste by protecting starch granules and improving the shear resistance of rice flour paste [141]. One study found that rice protein hydrolyzate had the highest gel hardness under acidic conditions. Thus, the interaction of protein with rice flour starch is different under different pH conditions. Therefore, rice flour products that meet target gel properties can be designed by controlling the degree of protein hydrolysis and controlling pH [142].

## 5. Conclusions and Outlook

Starch-based foods are the main dietary source of human nutrition and energy. Protein, as the largest non-starch component in starch-based foods, has covalent and non-covalent interactions with starch. So, its influence on the structure, physicochemical, and digestive properties of starch cannot be ignored. At the same time, endogenous protein in natural grains also has these effects on grain starch.

Research on how protein affects starch primarily examines its short-range and long-range ordered structures, including crystalline and granular arrangements. Generally speaking, whether it is endogenous protein or exogenously added protein, the interaction with starch can lead to several outcomes: protein may promote the formation of a compact structure of starch granules, form physical encapsulation of starch granules, or create protein–starch complexes. Factors affecting starch–protein interaction involve processing conditions, food pre-processing methods, and additives. Studies have shown that these factors have a certain impact on starch–protein interaction, so more research will be needed in the future to find factors that need to be considered in the processing of different foods, from pre-processing to processing.

A starch compact structure, whose formation is caused by protein, does not easily absorb water and gelatinize, thereby delaying the gelatinization process of starch and inhibiting the hydration of starch. Proteins can cause changes in the elastic and viscous properties of starch gels and reduce starch pseudoplasticity. The interaction of protein and starch contributes to the formation of a more continuous starch gel network structure, which in turn contributes to the good firmness and chewiness of starch-based foods. Furthermore, the interaction between protein and starch inhibits the cooling and rearrangement of gelatinized starch molecular chains, thereby delaying the aging of starch and helping the storage of starch-based foods. Finally, due to the physical encapsulation of starch by protein, the interaction with starch promotes the formation of starch compact structures and the competitive attraction of protein to α-amylase, such that the digestive properties of starch are effectively inhibited. Thus, the presence of protein helps to delay the production of blood sugar in starch-based foods. The favorable effects of protein starch have been verified in various staple food products (bread, steamed bread, noodles, rice flour, etc.).

In the future, the influence of protein on the multiscale structure of starch can be further studied to systematically analyze the relationship between the multiscale structure, protein type, and content. At the same time, the research methods for starch–protein interaction need further development. Finally, in industrial production, the interaction of other factors (the influence of processing conditions and other food components) should also be considered to appropriately formulate nutrient-rich, easy-to-process, starch-based foods. In addition, studies of the starch properties of endogenous proteins can be used to target grain varieties with specific protein content.

## Figures and Tables

**Table 1:** Related research on thermal characteristics and pasting properties of cereal starch significantly affected by endogenous proteins.

Type of Grain	Types of Protein Removed	Effects of Protein Removal on the Thermal Properties of Starch	References
Highland barley	Total endogenous protein	To and ΔH↑ Tc and Tc-To↓	[17]
Rice	SGAPs	To, Tp, Tc and gelatinization viscosity↓	[83]
Rice	SGCPs	To, Tp, Tc and FV↓	[66]
Wheat	SGSP	To and Tp↑	[85]
Sorghum	Total endogenous protein	Gelatinization temperature↓	[86]
Adlay seed	Total endogenous protein	ΔH, PV and BV↑ Gelatinization temperature and SV↓	[67]
Foxtail millet	Total endogenous protein	PV and ΔH↑ Gelatinization temperature↓	[87]

**note: ↑: Indicates an increase in [ ]. ↓: Represents a decrease in [ ].**

**Table 2:** Research on the digestive properties of grain starch significantly affected by endogenous proteins.

Type of Removal Protein	Research Object	Effects of Endogenous Proteins on Starch Digestion (Hydrolysis) Properties	Possible Causes of Effects on Starch Digestive Properties	References
SGAPs	Corn	Hydrolysis of corn-based gelatinized starch↓	SGAPs have stronger interactions with corn starch (hydrogen bonding, hydrophobic and electrostatic interactions) than storage proteins	[21]
Total endogenous protein	Spaghetti	Digestibility coefficient↓ Protection for long amylopectin↑	Physical interaction (entanglement or H-bonding) between protein and alpha-amylase prevents alpha-amylase from catalyzing starch hydrolysis.	[118]
Total endogenous protein	Rice	Inhibits digestibility of rice starch↑	Physical barrier function and ordered structure between protein and starch inhibit starch hydrolysis	[33]
Total endogenous protein	High Amylose Noodles	Inhibits digestibility of noodles with high amylose content↑	Gluten network physically entraps starch granules	[23]
Total endogenous protein	Adlay seed	Digestibility of adlay seed starch↓	Physical package of adlay seed starch	[68]
Quantitative protein removal	Rice	The more protein is removed, the lower the starch digestion.	Protein–starch interactions inhibit starch digestibility	[119]
Total endogenous protein	Buckwheat	Inhibits digestibility of buckwheat flour↓	The encapsulation of buckwheat starch granules by buckwheat protein limits the enzymatic hydrolysis of amylase	[120]
Total endogenous protein	Barley	Digestibility of rice flour↑	Enzyme binds with water-insoluble proteins and with starch granules, leading to reduced starch hydrolysis	[121]
SGAPs and storage protein	Corn flour	Removal of SGAPs is more inhibitory to corn starch hydrolysis than storage protein removal.	SGAPs interact strongly with starch chains through hydrogen bonding, hydrophobicity, and electrostatic interactions, whereas storage proteins strongly interact only through hydrogen bonding	[21]

**note: ↑: Indicates an increase in [ ]. ↓: Represents a decrease in [ ].**

**Table 3:** Research on the digestive characteristics of grain starch significantly affected by exogenous Proteins.

Exogenously Added Protein Species	Research Object	Effects on Starch Digestion (Hydrolysis) Properties	Possible Reasons Affecting Starch Digestion Properties	References
Durum wheat protein	Spaghetti	Starch digestion↓	Gelatinized pasta with higher protein content has more extensive cohesive network structure	[115]
Rice protein during digestion	Rice starch	α-amylase activity↓	After protein hydrolysis, rice starch has a more ordered aggregation structure	[117]
Whey protein isolate	Potato starch	Digestibility of potato starch↓	Interaction forces between whey protein isolate and potato starch (hydrophobic interactions, hydrogen bonding and inhibition of enzymatic activity)	[75]
Glutaminase-modified gluten and gliadin	Potato starch	Digestibility decreases with the increase in added protein concentration; RS↑	The addition of protein leads to an increase in intramolecular hydrogen bonds in starch	[122]
Whey protein isolate	Corn starch	Corn RSD content↑	Whey protein isolate forms a physical barrier around corn starch granules	[30]
Natural, denatured and hydrolyzed vegetable proteins from soy and pea proteins	Wheat starch	RDS content of wheat starch↑	Protein wrapping or embedding inside starch granules	[123]
Rice protein hydrolyzate	Wheat starch	Digestibility of wheat starch↓	The addition of rice protein promotes the formation of short-range ordered structure of wheat starch molecular chain, and the peptide chain is closely combined with the hydrogen of the -OH group of starch molecule	[22]

**note: ↑: Indicates an increase in [ ]. ↓: Represents a decrease in [ ].**

## Data Availability

No new data were created or analyzed in this study. Data sharing is not applicable to this article.
